# Hemp Fiber and Expanded Perlite-Incorporated Lightweight Inorganic Polymer Mortars: Mechanical, Thermal Insulation, High-Temperature Resistance, Microstructural Characteristics, and Life Cycle Assessment

**DOI:** 10.3390/polym18050653

**Published:** 2026-03-07

**Authors:** Brial Asif Hayi Paka, Turan Şevki Köker, Ezgi Orklemez, Guy Patrick Bikoula Onono, Ugur Durak, Serhan Ilkentapar, Okan Karahan, Cengiz Duran Atis

**Affiliations:** 1 Civil Engineering Department, Graduate School of Natural and Applied Sciences, Erciyes University, Kayseri 38030, Türkiye; tskoker@nny.edu.tr (T.Ş.K.); bikoulapatrick7@gmail.com (G.P.B.O.); 2 Construction Insulation Technology Program, Nuh Naci Yazgan University, Kayseri 38080, Türkiye; 3 Civil Engineering Department, Erciyes University, Kayseri 38030, Türkiye; orklemezezgi@gmail.com (E.O.); ugurdurak@erciyes.edu.tr (U.D.); serhan@erciyes.edu.tr (S.I.); okarahan@erciyes.edu.tr (O.K.); cdatis@erciyes.edu.tr (C.D.A.)

**Keywords:** geopolymer, fly ash, hemp fiber, perlite aggregate, life cycle assessment

## Abstract

In this study, lightweight geopolymer mortars with low environmental impact, high thermal insulation performance, and strong resistance to elevated temperatures were developed. Fly ash, expanded perlite, and bio-based hemp fibers were employed as the binder, aggregate, and reinforcement, respectively. Hemp fibers were prepared in lengths of 1, 2, and 3 cm and incorporated into the mixtures at dosages of 0.50%, 0.75%, and 1.00% by weight of binder. Sodium hydroxide was used as the activator, and specimens were heat-cured at 90 °C for 24–48–72 h. The workability, unit weight, UPV, flexural, and compressive strength of the geopolymer mortars were determined. In addition, thermal conductivity, high-temperature resistance, microstructural characteristics, and environmental impacts of selected mixtures were evaluated. The results demonstrated that lightweight geopolymer mortars could be successfully produced using expanded perlite aggregate and that hemp fibers significantly enhanced mechanical performance up to 48% at one day. Moreover, fiber reinforcement improved thermal insulation capability by up to 5.5% and high-temperature resistance. FESEM, EDX, elemental mapping, and XRD analyses supported the mechanical and physical findings through detailed microstructural evidence. Furthermore, LCA results revealed that fiber incorporation improved the environmental performance of geopolymer mortars, resulting in approximately a 21% reduction in global warming potential compared with the reference mixture.

## 1. Introduction

Cement production accounts for approximately 5–8% of global carbon dioxide emissions and therefore contributes significantly to global warming. Consequently, as the construction sector plays a critical role in climate change, researchers aim to mitigate global warming by developing alternative binders to conventional Portland cement [1,2,3,4,5,6,7,8]. To achieve this purpose, geopolymer-based binder systems have emerged as promising inorganic alternatives to traditional Portland cement due to their favorable mechanical properties, superior resistance to aggressive environments, and significantly lower environmental impacts. Geopolymer binders can be produced using industrial by-products such as fly ash, ground granulated blast furnace slag, and silica fume, as well as natural pozzolanic materials. In particular, the use of industrial by-products, such as fly ash, in geopolymer binders has been shown to provide not only satisfactory mechanical performance but also a substantial reduction in environmental impacts, as reported in numerous studies [3,9,10,11,12,13,14,15,16].

Geopolymer materials represent a novel class of silica–alumina-based binders formed through a unique reaction mechanism involving dissolution, depolymerization, and polymerization processes. Compared to cement-based construction materials, geopolymer systems can reduce carbon emissions by approximately 20%, thereby offering a more environmentally responsible alternative for sustainable construction practices [17,18]. Solid aluminosilicate-based materials activated with alkaline solutions undergo geopolymerization, yielding high-performance, low-carbon, and environmentally friendly construction materials. This process also facilitates the utilization of various solid wastes and industrial by-products, thereby contributing significantly to waste valorization and reducing carbon emissions [19].

In recent years, increasing attention has been directed toward high-performance thermal insulation materials that contribute to energy savings and reductions in carbon emissions, in line with sustainable development goals. While organic insulation materials commonly used in the construction sector have certain limitations, lightweight, porous, inorganic insulation materials are increasingly preferred for their superior thermal insulation performance. In this context, geopolymers, which exhibit outstanding thermal insulation and high-temperature resistance, are expected to play a significant role in future insulation and energy-efficient building applications [20,21,22,23,24,25].

One of the primary approaches for improving thermal insulation performance in concrete or mortar systems is the incorporation of lightweight aggregates. Among these aggregates, expanded perlite is one of the most commonly used materials. Perlite is a volcanic, glassy, and highly porous rock found in various regions worldwide, including Türkiye, Japan, China, and Hungary [26,27,28].

Raw perlite, which has approximately 7 billion tons of reserves worldwide and is extracted by open-pit mining, can expand to nearly 20 times its original volume when subjected to thermal treatment, making it suitable for lightweight concrete construction requiring acoustic and thermal insulation [29,30]. The use of both raw and expanded perlite in concrete and mortar applications has been widely demonstrated in previous studies. In particular, its effectiveness in geopolymer systems for reducing thermal conductivity and improving high-temperature resistance has been clearly reported in the literature [31,32,33].

Another practical approach to reducing environmental impacts in the construction sector is the development of bio-based concrete reinforced with plant fibers such as hemp. Hemp fiber, owing to its high mechanical performance and environmentally friendly nature, can be utilized in low-carbon concrete design to enhance toughness and durability. In addition, hemp acts as a carbon-sequestering material, absorbing more CO_2_ from the atmosphere during its growth and service life than it emits during its production and use stages, thereby contributing to reduced carbon emissions. Due to its carbon storage capability, hemp-based concrete is considered one of the carbon-negative construction materials. Previous studies on hemp-lime concrete have demonstrated that it can remove more greenhouse gases from the atmosphere than it emits over its life cycle [34,35,36,37,38,39,40,41]. Filazi et al. investigated hemp fiber-reinforced geopolymer composites. They reported that increasing fiber content significantly improved thermal insulation performance but led to reductions in compressive and flexural strength, highlighting a trade-off between mechanical and thermal properties [42]. Alcan et al. demonstrated that hemp fiber-reinforced RHA-based geopolymer markedly enhanced the mechanical and durability performance of kaolin clay and provided notable environmental advantages [34]. Galzerano et al. investigated hemp fiber grid reinforcement in lightweight geopolymer foams and reported improved flexural performance, non-brittle mechanical behavior, good fiber–matrix bonding, and preserved thermal stability without adverse effects on the physical properties of the composites [43]. Narattha et al. investigated Class C fly ash–based geopolymer composites incorporating AlCl_3_ and KOH-treated hemp shiv as a lightweight aggregate and reported that surface treatment enhanced geopolymerization, improved flexural strength and fracture toughness, and reduced thermal conductivity, although compressive strength and bulk density decreased with increasing hemp shiv content [44].

A review of the literature indicates that studies on the use of hemp fibers in geopolymer systems remain very limited. Also, studies on lightweight geopolymer composites with low environmental impact, high thermal insulation performance, and strong resistance to elevated temperatures were scanty. This study aims to develop sustainable mortar systems using fly ash–based geopolymer binders, 100% expanded perlite aggregates, and bio-based hemp fibers. Therefore, in the experimental program, lightweight hemp fiber-reinforced geopolymer mortars were produced by incorporating hemp fibers with lengths of 1, 2, and 3 cm at dosages of 0.50%, 0.75%, and 1.00% by weight into fly ash–based geopolymer composites containing 100% expanded perlite aggregates. The physical and mechanical properties, thermal insulation performance, high-temperature resistance, microstructural characteristics, and life cycle assessments of the produced lightweight geopolymer mortars were comprehensively investigated.

## 2. Materials and Methods

### 2.1. Materials

#### 2.1.1. Fly Ash

**Figure 1:**
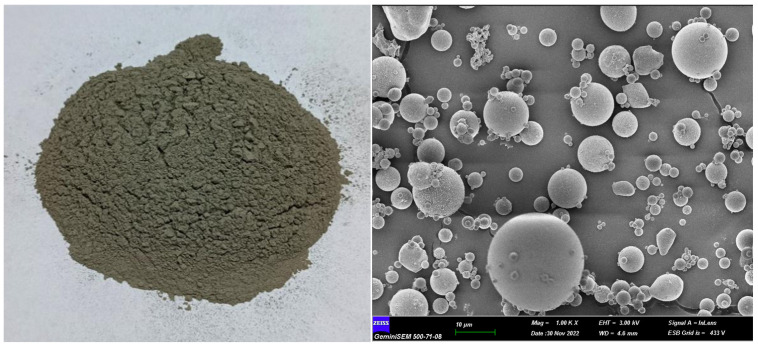
Physical appearance of fly ash and its SEM microstructure (1000×).

In this study, fly ash supplied from the Sugözü Thermal Power Plant located in Adana, Türkiye, was used as the binding material. The fly ash, with a density of 2.13 g/cm^3^, and its physical appearance and FESEM micrographs are presented in Figure 1. According to ASTM C618, the fly ash was classified as Class F, containing a total of 84.7% of SiO_2_, Al_2_O_3_, and Fe_2_O_3_. The detailed chemical composition is provided in Table 1.

#### 2.1.2. Expanded Perlite

Expanded perlite, a volcanic-origin material with high silica content and an amorphous structure, was used as aggregate in the production of lightweight geopolymer mortars in this study. When heated to approximately 850–900 °C, perlite undergoes significant volumetric expansion, forming a low-density, highly porous structure.

**Figure 2:**
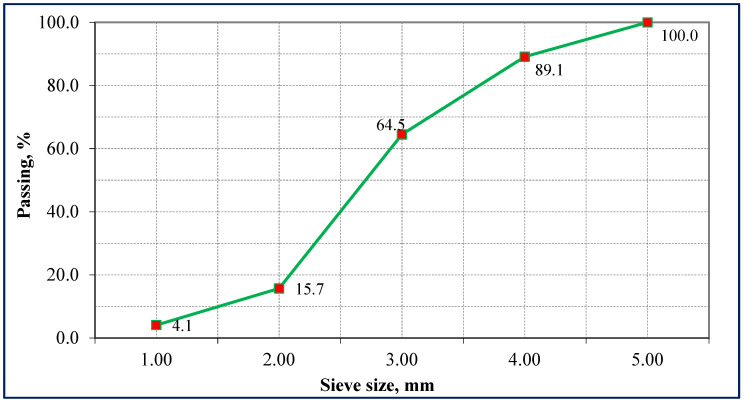
Sieve analysis of expanded perlite.

**Figure 3:**
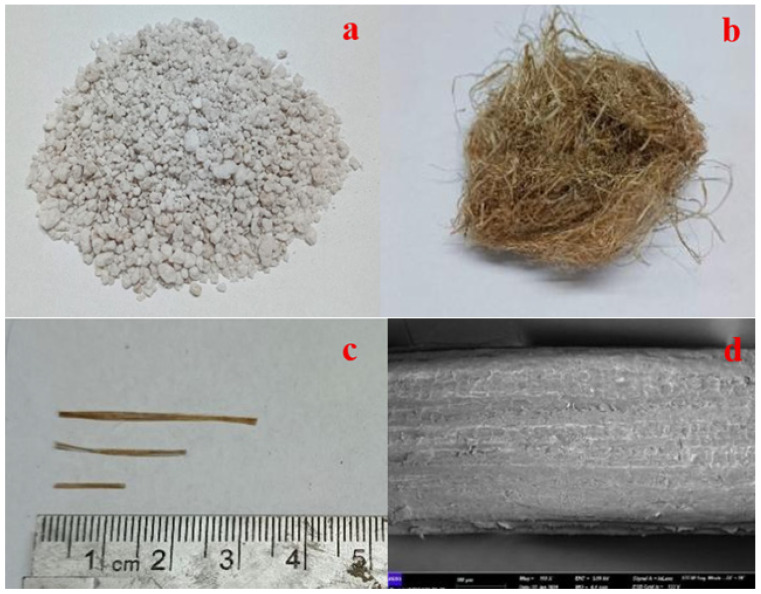
Physical appearance of perlite (**a**), hemp (**b**), and cut hemp fibers (**c**). SEM microstructure of hemp fiber (**d**) (100×).

The expanded perlite employed in this study had a bulk density of 90–150 kg/m^3^. Its sieve analysis is given in Figure 2, and its physical appearance is shown in Figure 3a. In building and material engineering applications, perlite is widely preferred to reduce the unit weight of composites and to improve thermal insulation performance. Moreover, due to its porous surface morphology, perlite provides good interfacial compatibility with fibers and the binder matrix in composite systems [45,46].

#### 2.1.3. Hemp Fiber

Hemp fiber, obtained from the Cannabis sativa plant, is a natural fiber widely preferred in engineering applications due to its high tensile strength and low biodegradability. In this study, hemp fibers were incorporated at three different lengths (1 cm, 2 cm, and 3 cm) to investigate their effect on the mechanical performance of geopolymer mortar. For each fiber length, three different dosages corresponding to 0.5%, 0.75%, and 1.0% by weight of binder were applied. The images of the hemp fibers used in this study are presented in Figure 3b–d).

#### 2.1.4. Superplasticizer (SP)

A polycarboxylate ether-based high-range water-reducing admixture was used to improve the workability and flowability of the mortar mixtures. According to previous studies, the use of PCE-based superplasticizers in geopolymer mixtures enhances particle dispersion, reduces porosity, and improves the mechanical strength and durability of the mortars [3,33].

#### 2.1.5. Alkali Activator

In this study, 99% pure sodium hydroxide (NaOH) was used as the alkaline activator. The alkaline solution employed in the mortar mixtures was prepared 24 h before casting to provide a Na^+^ content equivalent to 10% of the binder mass. Alkaline activators play a fundamental role in initiating and governing the geopolymerization process by promoting the dissolution of aluminosilicate phases present in fly ash.

Previous studies indicate that the selected NaOH concentration can yield optimal mechanical performance in geopolymer systems [16,47,48,49]. The chemical composition of the NaOH used in this study is presented in Table 2.

### 2.2. Methods

Fibers are widely used as reinforcement elements in cementitious and geopolymer systems due to their ability to enhance tensile strength, restrict microcrack development, and improve ductility. Moreover, natural fibers such as hemp contribute to sustainability owing to their renewable origin and low environmental impact. To clearly and reliably evaluate the influence of hemp fibers on geopolymer mortars, the binder type, alkaline activator content, and aggregate amount were kept constant in all mixtures. In contrast, only fiber length and dosage were considered variables. This experimental design ensured that the observed variations in performance could be directly attributed to the hemp fiber characteristics. The mixture proportions are presented in Table 3. The mixture codes were defined according to fiber length and dosage. The letter “H” represents hemp fiber, the first number indicates the fiber length (1, 2, or 3 cm), and the second value denotes the fiber dosage (0.50%, 0.75%, or 1.00% by weight of binder). For instance, H2-0.75 corresponds to a mixture containing 2 cm hemp fibers at a dosage of 0.75%. Hemp fibers were cut into lengths of 1, 2, and 3 cm and incorporated into the geopolymer mortar mixtures at dosages of 0.50%, 0.75%, and 1.00% by weight of binder.

During the specimen production process, fly ash and hemp fibers were first introduced into the mixing container and dry-mixed until a homogeneous powder blend was achieved. Subsequently, the sodium hydroxide–water solution, which had been prepared 24 h in advance and allowed to cool to room temperature, was added to the mixture. The mixture was then placed in a laboratory mortar mixer and mixed for 30 s at low speed (140 rpm). Following this stage, expanded perlite aggregate was added, and the mixing process was continued for an additional 60 s at low speed (140 rpm). The mixer was then stopped, and any material adhering to the sides of the mixing container was carefully scraped and collected at the center to ensure uniformity. During mixing, a polycarboxylate ether–based superplasticizer was incorporated to compensate for the high water absorption capacity of the expanded perlite and the combined effects of fiber length and dosage on workability. After the addition of the superplasticizer, the mixture was further mixed for 90 s at high speed (280 rpm) to obtain a uniform fresh mortar. However, despite the use of the superplasticizer at the manufacturer’s maximum recommended dosage, the target workability could not be fully achieved. Therefore, the mixing water content was adjusted to ensure comparable consistency and flowability across all mixtures. Although minor adjustments to the mixing water content were made to achieve comparable workability, these modifications did not alter the Na^+^ ratio. Upon completion of the mixing procedure, the freshly prepared mortar mixtures were placed into 40 × 40 × 160 mm steel molds using a vibration table.

Fresh mortars were heat-cured at 90 °C for 24, 48, and 72 h. The workability of the fresh geopolymer mortars was evaluated using the mini flow table test in accordance with TS EN 1015-3 [50]. The unit weights of the hardened specimens were determined in accordance with ASTM C642 [51], and the average of three measurements was reported for each mixture. Ultrasonic pulse velocity (UPV) measurements were carried out in accordance with TS EN 12504-4 [52]. The measurements were performed along the longitudinal axis of 40 × 40 × 160 mm^3^ prismatic specimens after heat curing and cooling to room temperature. For each mixture series, three specimens were tested, and the average UPV values were reported.

Flexural and compressive strength tests were conducted in accordance with TS EN 1015-11 [53]. After curing at 90 °C for 24, 48, and 72 h, flexural strength tests were performed on three prismatic specimens measuring 40 × 40 × 160 mm^3^. The broken halves were subsequently used for compressive strength testing with 40 × 40 mm loading areas. The results were evaluated as the average of three flexural and six compressive strength measurements. Based on the mechanical performance results, the optimal heat-curing duration was determined to be 48 h, and all subsequent experiments were conducted with this curing period. Accordingly, geopolymer mortars containing 0.75% hemp fiber with fiber lengths of 1, 2, and 3 cm (H1-0.75, H2-0.75, and H3-0.75) were selected for the thermal conductivity and high-temperature resistance tests. In addition, detailed microstructural characterization, including field-emission scanning electron microscopy (FESEM), energy-dispersive X-ray spectroscopy (EDX), elemental mapping, and X-ray diffraction (XRD), as well as life-cycle assessment (LCA), was performed on the H3-0.75 mixture, which exhibited the highest mechanical strength.

Thermal conductivity measurements were performed on three specimens in accordance with TS EN 12667 [54]. In the high-temperature resistance tests, the specimens were heated to 300 °C, 600 °C, and 900 °C for 1 h then allowed to cool naturally to room temperature. After cooling, weight loss, ultrasonic pulse velocity, and flexural and compressive strength values were determined.

Microstructural analyses were conducted on specimens cured at 90 °C for 48 h using FESEM, EDX, and elemental mapping techniques. Before analysis, the specimen surfaces were coated with gold–palladium, and examinations were performed using a Zeiss Gemini SEM 500 device. For XRD analysis, ground samples were sieved through a 63 µm mesh and analyzed using a Bruker AXS D8 diffractometer with a CuKα radiation source over a 2θ range of 5–60°.

**Figure 4:**
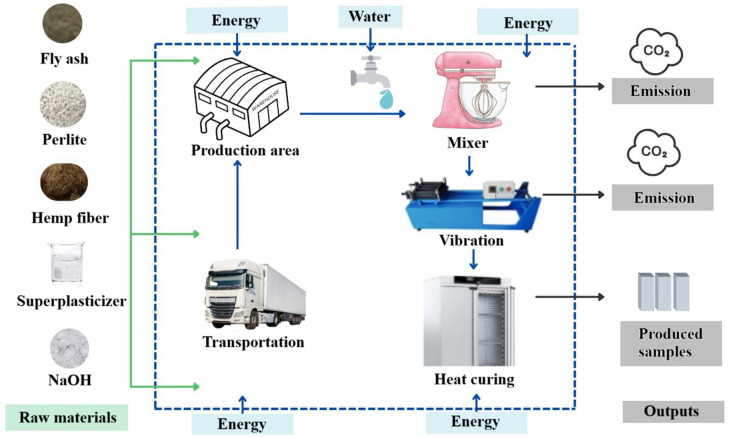
System boundaries.

Life cycle assessment was performed in accordance with ISO 14040 [55] and ISO 14044 [56], adopting a cradle-to-gate approach within the system boundaries shown in Figure 4. The functional unit was defined as 1 m^3^/MPa. Primary inventory data were obtained from local manufacturers, and energy consumption during production was monitored in real time. Environmental impact assessments were conducted using SimaPro with the Ecoinvent database, applying the CML-IA baseline method. The impact categories quantified included abiotic depletion (AD), abiotic depletion–fossil fuels (AD-FF), global warming potential (GWP), ozone depletion potential (ODP), human toxicity (HT), freshwater aquatic ecotoxicity (FAE), marine aquatic ecotoxicity (MAE), terrestrial ecotoxicity (TE), photochemical oxidation (PO), acidification potential (AP), and eutrophication potential (EP).

## 3. Results and Discussion

### 3.1. Workability

**Figure 5:**
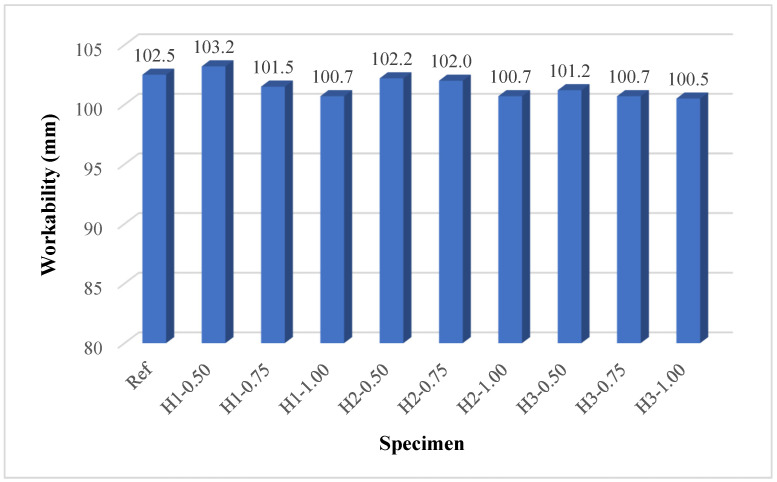
Workability results.

The workability test results presented in Figure 5 clearly demonstrate the influence of hemp fiber length and content on the fresh mortar behavior, since the fly ash and perlite contents were kept constant in all mixtures. While the reference mixture (Ref) exhibited a spread diameter of 102.5 mm, the spread diameter of the fiber-reinforced mixtures generally decreased with increasing fiber content [57]. For instance, the H1-0.50 mixture showed a spread diameter of 103.2 mm, which was very close to and even slightly higher than that of the reference mixture; however, when the fiber content was increased to 1.0% in the H1-1.00 mixture, the spread diameter decreased to 100.7 mm. A similar trend was observed for the 2 cm and 3 cm fiber lengths, where the H2-1.00 and H3-1.00 mixtures yielded the lowest spread diameters of 100.7 mm and 100.5 mm, respectively. This behavior indicates that the increase in fiber content leads to higher internal friction due to the enlarged fiber surface area and increased contact points within the mixture, thereby restricting the flowability of the fresh mortar. The reduction in spread diameter with increasing fiber content is consistent with previous studies [57,58,59,60]. When the effect of fiber length was examined, a slight but systematic reduction in spread diameter was observed with increasing fiber length at the duplicate fiber content. For example, at 0.75% fiber content, the spread diameter decreased from 101.5 mm for H1-0.75 to 102.0 mm for H2-0.75 and further to 100.7 mm for H3-0.75. Similarly, at 1.0% fiber content, the H1-1.00 and H2-1.00 mixtures exhibited spread diameters of 100.7 mm, whereas the H3-1.00 mixture had the lowest spread diameter of 100.5 mm. This trend reveals that longer fibers tend to entangle more easily, increasing the risk of agglomeration and further limiting the flow of fresh mortar.

Although the mixing water content was deliberately increased with higher fiber dosages, the observed reduction in workability highlights the dominant influence of the high specific surface area and water absorption capacity of hemp fibers on the rheological behavior of fresh mortars. Nevertheless, the fact that all fiber-reinforced mixtures exhibited spread diameters within the range of 100–103 mm indicates that they could still be produced within an acceptable workability range and that the applied water adjustment strategy was sufficient for practical applications. Overall, the most balanced workability performance was achieved at lower fiber contents and shorter fiber lengths. In contrast, the combined increase in fiber length and dosage exerted a limiting effect on the flowability of the fresh geopolymer mortars.

### 3.2. Unit Weights

**Figure 6:**
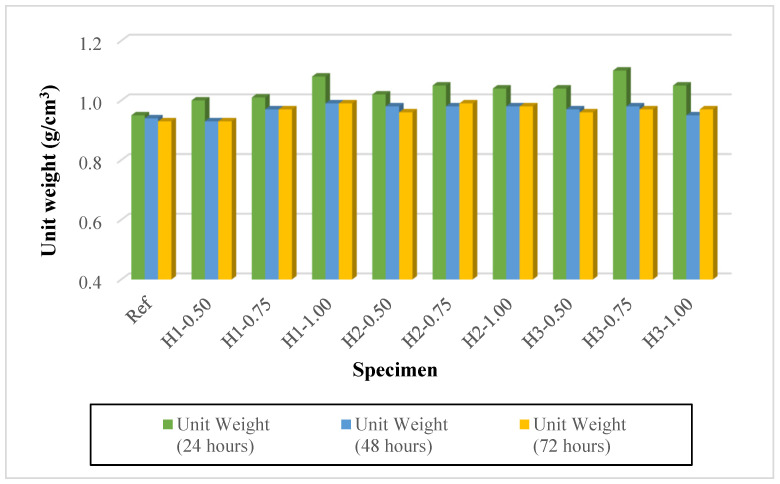
Unit weights of mortars.

Owing to the use of expanded perlite aggregate, lightweight geopolymer mortars with very low unit weights were successfully produced in all mixtures, and the influence of thermal curing at 90 °C for 24, 48, and 72 h was clearly reflected in the unit weight results (Figure 6). In the reference mixture (Ref), the unit weight gradually decreased from 0.95 on the first day to 0.93 on the third day. In contrast, the fiber-reinforced mixtures generally exhibited higher unit weight values on the first day (e.g., H1-1.00: 1.08; H3-0.75: 1.10). This behavior can be attributed to the increased water demand required to maintain comparable workability after fiber addition, as well as to the water-retaining and entrapping capacity of hemp fibers, which results in a higher amount of free and bound water within the fresh and early-cured matrix. As the thermal curing duration was extended to 48 and 72 h, the unit weights of all mixtures decreased, which is associated with accelerated moisture loss under the 90 °C environment and rapid release of water from both the porous perlite structure and the geopolymer matrix. Accordingly, the most pronounced reductions generally occurred between 24 and 48 h, whereas the changes between 48 and 72 h were limited, leading to a plateau-like behavior in many mixtures (e.g., H1-0.75: 1.01 → 0.97 → 0.97; H1-1.00: 1.08 → 0.99 → 0.99). With respect to fiber content, increasing fiber dosage at the same fiber length resulted in higher unit weights on the first day (H1 series: 1.00 < 1.01 < 1.08). This trend can be explained by the higher water requirement associated with increased fiber dosage, as well as the additional internal friction and porous structure induced by the fibers. Even after 72 h of curing, mixtures with higher fiber content generally maintained slightly higher unit weights (0.97–0.99) than the reference mixture, which may be related to changes in fiber arrangement within the matrix and the resulting porosity.

The effect of fiber length was more pronounced at low and moderate fiber contents. At 0.50% and 0.75% fiber dosages, increasing the fiber length from 1 cm to 3 cm led to a systematic increase in the first-day unit weight values (H1–H2–H3)-0.50: 1.00 → 1.02 → 1.04; H1–H2–H3)-0.75: 1.01 → 1.05 → 1.10). This behavior indicates that longer fibers tend to increase the water demand and water retention in the fresh matrix due to their greater tendency to entangle and agglomerate. In conclusion, although the lightweight character achieved through expanded perlite was successfully preserved, the fiber dosage and fiber length of hemp fibers (along with the corresponding water adjustment) increased the unit weight, particularly at early ages. As the curing duration at 90 °C increased, the unit weights decreased due to moisture loss, and after 48 h, the variations became considerably smaller, approaching a more stable level.

### 3.3. Ultrasonic Pulse Velocity (UPV)

**Figure 7:**
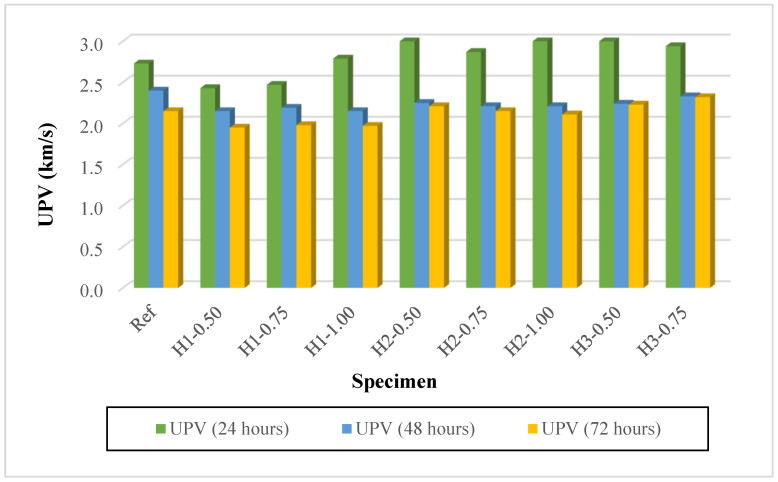
UPV results of mortars.

The ultrasonic pulse velocity (UPV) results are presented in Figure 7. For lightweight geopolymer mortars produced with expanded perlite, UPV serves as a highly sensitive indicator of matrix continuity, pore structure, and microcrack formation, clearly reflecting the variations associated with hemp fiber length and content. A general trend observed in all mixtures is the significant decrease in UPV values as the thermal curing duration at 90 °C was extended from 24 to 48 and 72 h (Ref: 2.73 → 2.40 → 2.15 km/s). This reduction can be attributed to rapid moisture loss during thermal curing, the resulting shrinkage stresses and microcrack development, and the increasing obstruction of wave propagation in porous perlite-based systems. In fiber-reinforced mixtures, the effect of fiber length is pronounced. In the H1 series containing 1 cm fibers, UPV values generally remained lower than those of the reference mixture (third day: 1.95–1.98 km/s). This behavior may be explained by the fact that although short fibers provide dispersed reinforcement within the matrix, the increased water demand and the discontinuities at the fiber–matrix interface weaken ultrasonic wave transmission.

In contrast, mixtures containing 2 cm and 3 cm fibers (H2 and H3 series) exhibited higher UPV values, particularly in the first-day measurements (e.g., H2-0.50: 3.10 km/s; H3-0.50: 3.08 km/s), suggesting that longer fibers may enhance early-age crack bridging and microstructural continuity. Regarding fiber content, increasing the fiber dosage at the same fiber length did not produce a unidirectional, continuous effect on UPV values. However, the third-day UPV values of the H2 and H3 series remained relatively high, within the range of 2.11–2.32 km/s. In particular, the H3-0.75 and H3-1.00 mixtures reached UPV values of 2.32 and 2.31 km/s after 72 h, indicating that longer fibers can partially preserve ultrasonic wave transmission paths by restricting the propagation of microcracks induced by thermal curing. Taken together, the UPV results suggest that increasing the heat-curing duration reduces wave velocity across all mixtures. Nevertheless, increasing the hemp fiber length to 2–3 cm better preserves microstructural continuity, particularly at extended curing durations, resulting in higher UPV values than in the H1 series.

### 3.4. Flexural Strength Results

**Figure 8:**
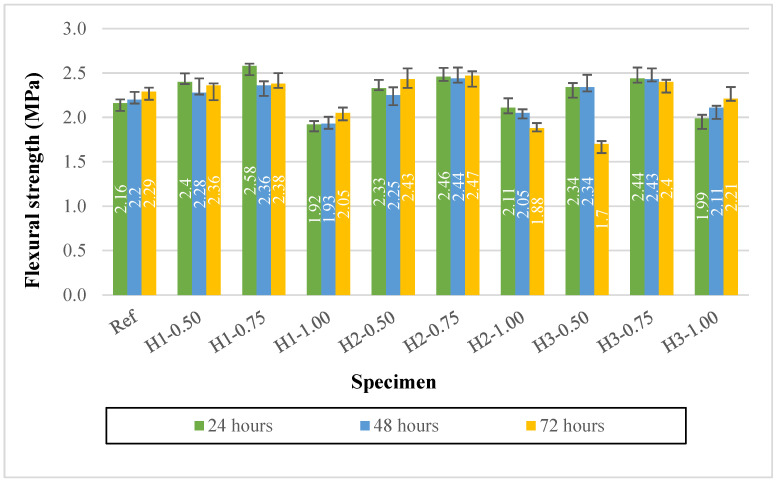
Flexural strengths of mortars.

The effect of hemp fiber dosage and length on the flexural strength of lightweight geopolymer mortars produced with expanded perlite is presented in Figure 8. The standard deviation was calculated for each flexural strength data point. The average standard deviation for the flexural strength results was approximately 5%, with a maximum value of about 9%. While the flexural strength of the reference mixture (Ref) increased slightly but steadily from 2.16 to 2.29 MPa between 1 and 3 days of heat curing, the mixtures containing fibers generally exhibited improved flexural strength at low and moderate fiber contents.

In particular, in the series containing 1 cm fibers, the H1-0.50 and H1-0.75 mixtures reached flexural strength values of 2.36 and 2.38 MPa on the third day, respectively, exceeding that of the reference mixture. This improvement can be attributed to the ability of short fibers to be more homogeneously dispersed, thereby bridging microcracks and enhancing ductility and energy absorption capacity under flexural loading. In contrast, when the fiber content was increased to 1.0% in the H1-1.00 mixture, the flexural strength remained low at all ages (1.92–2.05 MPa). This behavior indicates that the increased water demand required for workability, combined with the tendency of fibers to agglomerate and create interfacial discontinuities at high fiber contents, can generate weak zones in the matrix, adversely affecting flexural performance.

As fiber length increased, the trend was not unidirectional, underscoring the importance of an optimal fiber dosage. In the 2 cm fiber series, the H2-0.50 and, especially, the H2-0.75 mixtures exhibited the most balanced and highest performance; the H2-0.75 specimen reached 2.47 MPa on the third day, one of the highest values among all mixtures. This result suggests that 2 cm fibers are sufficiently long to provide effective crack bridging while remaining short enough to avoid excessive entanglement and agglomeration. However, when the fiber content was increased to 1.0% for the 2 cm fibers (H2-1.00), the flexural strength decreased to 1.88 MPa on the third day and continued to decline with age. This behavior is consistent with increased fiber interaction, void formation, and weakened interfacial bonding at high fiber dosages.

In the 3 cm fiber series, the behavior appears more sensitive. The sharp decrease in flexural strength to 1.70 MPa on the third day for the H3-0.50 mixture indicates that long fibers, even at low dosages, can locally agglomerate and create discontinuities, enabling thermally induced microcracks to propagate through these weak regions. In contrast, the H3-0.75 mixture exhibited a high and stable flexural strength of 2.40 MPa on the third day, while the H3-1.00 mixture, although showing a lower early-age strength, increased to 2.21 MPa by the third day.

Collectively, the results demonstrate that a moderate fiber content (0.75%) provides a more reliable optimum range for flexural strength in lightweight geopolymer mortars, whereas increasing the fiber content to 1.0% increases the water demand and complicates fiber dispersion, thereby posing a risk of reduced flexural performance.

### 3.5. Compressive Strength Results

**Figure 9:**
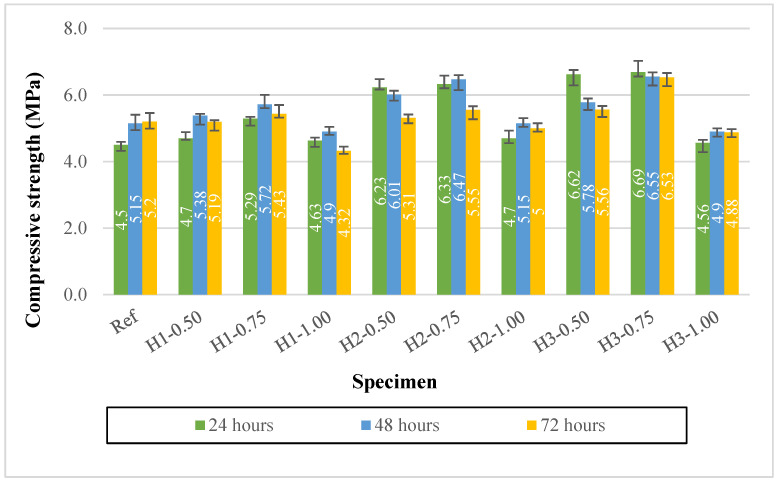
Compressive strengths of mortars.

The effects of hemp fiber length and dosage on the compressive strength of lightweight geopolymer mortars produced with expanded perlite aggregate are presented in Figure 9. The standard deviation was calculated for each compressive strength data point. The average standard deviation for the compressive strength results was approximately 4%, with a maximum value of about 7%. The compressive strength of the reference mixture (Ref) ranged between 4.5 and 5.2 MPa from 1 to 3 days. The incorporation of fibers exhibited two distinct trends depending on fiber length and dosage. At low and moderate fiber contents (particularly 0.50% and 0.75%), several mixtures showed noticeable strength improvements compared to the reference, whereas mixtures containing 1.00% fiber generally exhibited reduced or limited strength development.

This behavior is clearly observed in the 1 cm fiber series (H1). While the H1-0.75 mixture reached 5.43 MPa on the third day, exceeding the reference value, the compressive strength of the high-fiber-content H1-1.00 mixture decreased to 4.32 MPa, the lowest value. This result suggests that increasing the fiber dosage requires higher water content to maintain workability. Still, this higher water content promotes fiber agglomeration and interfacial discontinuities, thereby reducing matrix continuity and adversely affecting compressive strength.

With increasing fiber length, particularly in the 2–3 cm fiber series, higher early-age strengths were observed at low and moderate fiber contents. The H2-0.50 and H2-0.75 mixtures exhibited remarkable increases in compressive strength on the first day, reaching 6.23 and 6.33 MPa, respectively, compared to the reference mixture, and remained at comparable or higher levels on the third day (5.31–5.55 MPa). Among the 3 cm fiber mixtures, the H3-0.75 specimen showed the best performance, achieving the highest early-age strength of 6.69 MPa on the first day and maintaining the highest and most stable value of 6.53 MPa on the third day. This behavior indicates that, with an appropriate combination of fiber length and dosage, fibers can effectively restrict microcrack development, delay crack propagation under compression, and support load transfer within the matrix.

In contrast, at a fiber content of 1.00% (H2-1.00 and H3-1.00), the compressive strengths remained within the range of 4.56–5.00 MPa, highlighting that once the optimum fiber dosage is exceeded, the additional voids and weak interfacial regions introduced by the fibers become dominant, leading to a reduction in compressive strength. Regarding the effect of curing duration, the observation that the high strengths obtained on the first day did not increase proportionally on the second and third days, and even decreased in some mixtures (e.g., H2-0.50: 6.23–6.01–5.31; H3-0.50: 6.62–5.78–5.56), suggests that prolonged thermal curing at 90 °C may influence compressive behavior through rapid moisture loss and possible microcrack formation. This effect can be more pronounced in lightweight systems containing porous perlite.

Taken together, the results indicate that the most favorable fiber dosage for compressive strength is generally 0.75%. In terms of fiber length, 2–3 cm fibers at appropriate dosages (particularly H3-0.75) can significantly enhance compressive strength. However, increasing the fiber content to 1.00% introduces a risk of strength reduction due to higher water demand and microstructural discontinuities.

### 3.6. Elevated Temperature Test Results

Based on the mechanical strength results from the geopolymer mortar specimens, the mixtures with a fiber content of 0.75%, which exhibited the optimum performance, were selected, and elevated-temperature tests were conducted on specimens reinforced with hemp fibers 1, 2, and 3 cm in length.

#### 3.6.1. Weight Loss After Elevated Temperature Exposure

The weight losses measured after the elevated temperature tests are presented in Table 4. The results obtained from the selected specimens indicate that, in lightweight geopolymer mortars incorporating expanded perlite, the mass loss increased gradually with rising temperature, as expected, and that hemp fibers (at a fixed content of 0.75% in this stage) were able to influence the moisture transport and drying behavior, particularly at 300 °C. In the reference mixture (Ref), the weight losses at 300, 600, and 900 °C were 4.88%, 7.01%, and 8.18%, respectively, whereas lower losses were recorded for the fiber-reinforced mixtures at 300 °C (H1-0.75: 4.24%, H2-0.75: 4.54%, H3-0.75: 4.71%). This behavior can be attributed to the fibers’ ability to restrict early microcrack propagation and delay moisture release, as well as their potential to retain part of the water within the matrix. When the temperature was increased to 600 °C, the weight loss in all mixtures rose to approximately 7–8%, indicating that most of the free and chemically bound water had been removed, accompanied by dehydroxylation of the geopolymer gel and structural changes in the perlite-based porous system. At this temperature level, the fiber-reinforced mixtures did not exhibit a clear advantage over the reference mixture; on the contrary, slightly higher losses were observed for H2-0.75 (7.76%) and H3-0.75 (7.93%).

At 900 °C, the mass loss reached its maximum values, and the effect of fiber length became more pronounced. While H1-0.75 exhibited the lowest weight loss (7.36%), the mass loss increased with increasing fiber length (H2-0.75: 8.25%, H3-0.75: 8.75%). This trend can be explained by the decomposition and burning of longer fibers at high temperatures, which generate additional voids and a more connected pore network, thereby facilitating the release of volatile components. Briefly, the increase in mass loss with fiber length at 900 °C demonstrates that fiber length is a critical parameter for fire resistance. In contrast, at 300 °C, the fiber-reinforced mixtures maintained better microstructural integrity than the reference mixture, as reflected in their lower weight loss.

#### 3.6.2. UPV Results After Elevated Temperature Exposure

The ultrasonic pulse velocity (UPV) results obtained after elevated temperature exposure are presented in Table 5. UPV measurements after exposure to 900 °C were performed only after the specimens had completely cooled to room temperature. Before high-temperature exposure, the fiber-reinforced mixtures exhibited UPV values similar to or higher than those of the reference mixture (Ref: 2.15 km/s; H3-0.75: 2.32 km/s), suggesting that, at a fiber content of 0.75%, hemp fibers limited microcrack development and supported matrix continuity. When the temperature was increased to 300 °C, a pronounced decrease in UPV values was observed in all mixtures (approximately 20–25%), attributed to the removal of free and partially bound water, as well as to the formation of initial microvoids and thermal stresses that weakened wave transmission. At this stage, the fiber-reinforced mixtures exhibited values very close to, or slightly higher than, those of the reference mixture (e.g., H3-0.75: 1.81 km/s), suggesting that the fibers partially preserved the microstructural integrity at early temperature levels.

At 600 °C, the UPV values decreased to their lowest levels in all mixtures (approximately 1.52–1.59 km/s), pointing to a significant increase in microcrack density associated with dehydroxylation of the geopolymer gel, expansion of the perlite-based porous structure, and the onset of fiber degradation. The limited differences among mixtures at this temperature confirm that 600 °C represents a critical damage threshold for all compositions. In contrast, at 900 °C, the UPV values increased markedly in all specimens, reaching the range of 3.62–3.68 km/s. This recovery is attributed to matrix sintering, reorganization of glassy phases, and partial pore closure, resulting in a more continuous microstructure. The fact that the fiber-reinforced mixtures exhibited values similar to or slightly higher than those of the reference mixture indicates that, although the fibers were largely decomposed at this temperature, the remaining voids were partially compensated by matrix restructuring. In conclusion, the UPV results demonstrate that microstructural damage is dominant at 300 and 600 °C, whereas at 900 °C, the system undergoes a reorganization process that enhances wave transmission.

#### 3.6.3. Flexural Strength Results After Elevated Temperature Exposure

**Figure 10:**
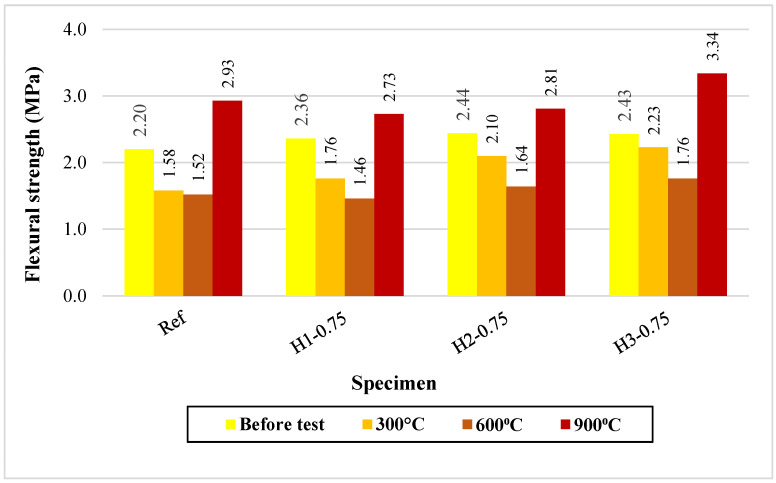
Flexural strengths of mortars after elevated temperature.

The flexural strength results obtained before and after elevated temperature exposure are presented in Figure 10. Before high-temperature exposure, the fiber-reinforced mixtures exhibited higher flexural strength values than the reference mixture (Ref: 2.20 MPa; H1-0.75: 2.36 MPa; H2-0.75: 2.44 MPa; H3-0.75: 2.43 MPa), confirming that the fibers improved the brittle matrix behavior through an effective crack-bridging mechanism. When the temperature was increased to 300 °C, the flexural strength of the reference specimen decreased from 2.20 to 1.58 MPa, whereas the fiber-reinforced mixtures, particularly those containing 2 cm and 3 cm fibers, preserved higher residual strengths. Specifically, H2-0.75 and H3-0.75 reached 2.10 and 2.23 MPa, respectively, exhibiting minor strength losses and outperforming the reference mixture after exposure to 300 °C. This finding suggests that at moderate temperatures, the fibers can effectively restrict microcrack propagation and maintain matrix continuity, thereby contributing to load transfer under flexural loading.

At 600 °C, a further reduction in flexural strength was observed in all mixtures (Ref: 1.52 MPa; H1-0.75: 1.46 MPa; H2-0.75: 1.64 MPa; H3-0.75: 1.76 MPa), suggesting an increase in microcrack density associated with dehydroxylation and degradation of the geopolymer binder phase, together with the accelerated thermal decomposition of hemp fibers. Nevertheless, the highest residual strength was still observed for H3-0.75 (1.76 MPa), implying that longer fibers provided partial crack bridging and crack control even within this critical temperature range. At 900 °C, a notable increase in flexural strength was observed in all mixtures (Ref: 2.93 MPa; H3-0.75: 3.34 MPa). This “partial recovery” is attributed to matrix sintering, reorganization of glassy phases, and partial pore closure, resulting in a more compact microstructure. The highest residual flexural strength achieved by H3-0.75 at this stage indicates that a fiber length of 3 cm at a dosage of 0.75% is the most effective option for improving post-fire mechanical performance.

Overall, the results demonstrate that the detrimental effect of temperature on flexural strength is dominant in the 300–600 °C range, while increasing fiber length—particularly to 2–3 cm—enhances the preservation of residual flexural strength. At 900 °C, however, microstructural reorganization enables a partial recovery of flexural strength in all mixtures.

#### 3.6.4. Compressive Strength Results After Elevated Temperature Exposure

**Figure 11:**
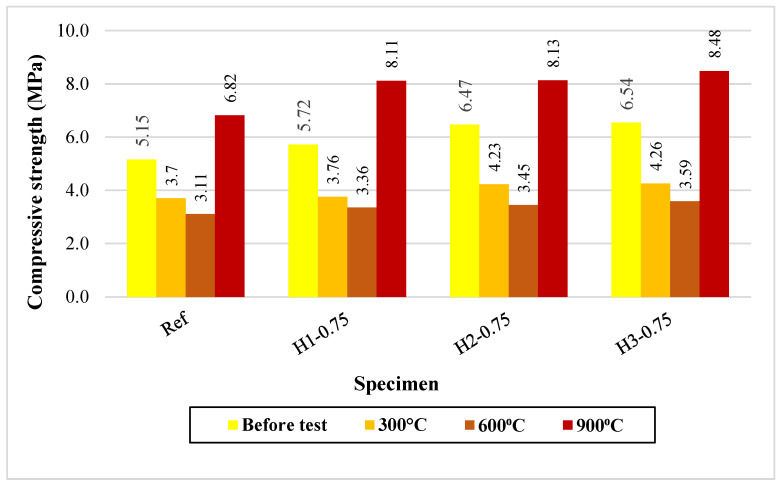
Compressive strengths of mortars after elevated temperature.

The compressive strength results obtained before and after elevated temperature exposure are presented in Figure 11. Before high-temperature exposure, the fiber-reinforced mixtures exhibited higher compressive strength values than the reference mixture (Ref: 5.15 MPa; H1-0.75: 5.72 MPa; H2-0.75: 6.47 MPa; H3-0.75: 6.54 MPa), revealing that the fibers enhanced matrix continuity by restricting microcrack propagation and increasing the load-bearing capacity of the lightweight system. When the temperature was increased to 300 °C, a pronounced reduction in compressive strength was observed in all mixtures (Ref: 3.70 MPa); however, the fiber-reinforced specimens maintained higher values than the reference mixture. In particular, the H2-0.75 and H3-0.75 mixtures, containing 2–3 cm fibers, retained compressive strengths of 4.23–4.26 MPa, suggesting that the crack-bridging effect of the fibers remained effective at this temperature level and limited thermally induced damage.

At 600 °C, a further decrease in compressive strength was recorded (Ref: 3.11 MPa; H1-0.75: 3.36 MPa; H2-0.75: 3.45 MPa; H3-0.75: 3.59 MPa), reflecting the combined effects of dehydroxylation and structural degradation of the geopolymer gel phase, together with the pronounced thermal decomposition of hemp fibers, which increased microcrack density and the formation of connected pores. Nevertheless, the increase in residual compressive strength with increasing fiber length, with H3-0.75 exhibiting the highest value, indicates that longer fibers provided more effective crack control and load transfer even under these severe conditions. At 900 °C, the compressive strength of all mixtures increased again and exceeded the pre-exposure levels (Ref: 6.82 MPa; fiber-reinforced mixtures: 8.11–8.48 MPa). This “strength gain” can be attributed to matrix sintering, reorganization of glassy phases, and the formation of a more compact microstructure at high temperatures [14,16,33]. The higher strength values of the fiber-reinforced mixtures compared with the reference mixture suggest that, although the fibers were consumed mainly at this temperature, their initial contribution to improved dispersion and crack control resulted in a microstructure more favorable for high-temperature-induced reorganization.

As a result, although increasing temperature in the range of 300–600 °C reduced the compressive strength of all mixtures, hemp fibers—particularly at lengths of 2–3 cm—effectively limited damage by providing higher residual strength. At 900 °C, all mixtures exhibited significant strength recovery, with the H3-0.75 mixture achieving the highest performance. Similar high-temperature stability and microstructural densification mechanisms have been documented in one-part geopolymer systems [61].

### 3.7. Thermal Conductivity Results

**Figure 12:**
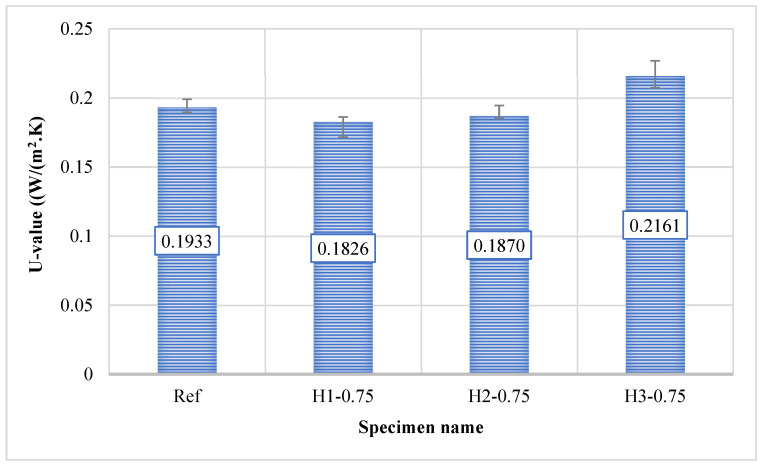
Thermal conductivity of mortars.

The thermal conductivity results presented in Figure 12 demonstrate that all mixtures, which were cured at 90 °C for 48 h before testing, exhibited low heat transfer characteristics due to the highly porous structure of the expanded perlite aggregate; however, at a hemp fiber content of 0.75%, fiber length significantly influenced the heat transfer mechanism in different ways. While the reference mixture (Ref) showed a thermal conductivity value of 0.1933 W/(m·K), lower values were obtained for the mixtures containing 1 cm and 2 cm fibers (H1-0.75: 0.1826 W/(m·K); H2-0.75: 0.1870 W/(m·K)). In the H1-0.75 mixture, the thermal conductivity coefficient decreased by approximately 5.5% relative to the reference, while in the H2-0.75 mixture, a reduction of about 3.3% was observed. This behavior suggests that the fibers introduced additional dispersed voids into the matrix and created more discontinuous heat-transfer paths, thereby enhancing the perlite’s insulation effect.

In contrast, the H3-0.75 mixture, incorporating 3 cm fibers, exhibited a noticeably higher thermal conductivity value of 0.2161 W/(m·K), exceeding that of both the reference and the other fiber-reinforced mixtures. The thermal conductivity coefficient of the H3-0.75 mixture increased by approximately 11.8% compared to the reference mixture. This increase can be attributed to the tendency of longer fibers to partially align and intertwine within the matrix, forming continuous fiber networks that may act as thermal bridges, as well as to local fiber agglomerations that generate denser regions facilitating heat flow. Consequently, even though the overall porosity of the system may increase, the formation of more connected heat transfer pathways can result in higher thermal conductivity. Overall, at a fiber content of 0.75%, the most favorable thermal insulation performance was achieved with the H1-0.75 mixture, while maintaining the fiber length within the range of 1–2 cm effectively reduced thermal conductivity; in contrast, a fiber length of 3 cm produced an adverse effect by increasing thermal conductivity in this study.

The thermal conductivity values obtained in this study demonstrate markedly superior insulation performance compared with those of conventional building materials. In the literature, the thermal conductivity of standard concrete is generally reported to be 0.6–3.3 W/(m·K), while lightweight concrete typically exhibits values of 0.2–1.9 W/(m·K) [62,63,64]. The values measured in this study, ranging from 0.1826 to 0.2161 W/(m·K), indicate that the developed geopolymer mortars not only outperform normal and lightweight concretes but are also comparable to, and in some cases superior to, pumice blocks in terms of thermal insulation performance. In particular, the H1-0.75 and H2-0.75 mixtures, with values of 0.18–0.19 W/(m·K), demonstrate that these materials are promising alternatives for applications requiring enhanced thermal insulation. These findings clearly show that the highly porous structure of the perlite aggregate, together with the lightweight, discontinuous nature of the geopolymer matrix, significantly restricts heat transfer paths, confirming that the developed system represents a potential construction material offering both structural functionality and improved energy efficiency.

### 3.8. Field Emission Scanning Electron Microscopy Analysis (FESEM)

**Figure 13:**
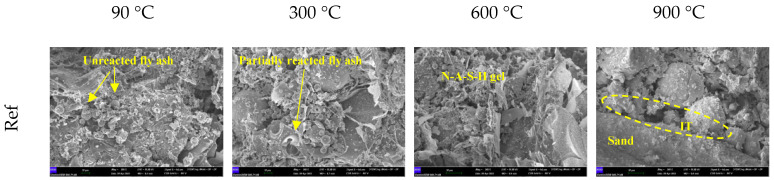

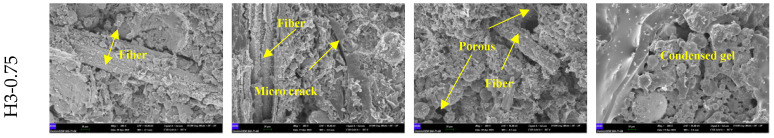
FESEM images of specimens.

The FESEM investigations were conducted on the H3-0.75 specimen, which exhibited the best compressive strength, and on the reference specimen; the obtained micrographs are presented in Figure 13. The specimens were first heat-cured at 90 °C for 48 h and then exposed to 300, 600, and 900 °C to examine microstructural transformations with increasing temperature.

In the reference specimen cured at 90 °C for 48 h, a considerable amount of unreacted fly ash spheres remained in the matrix, indicating that the geopolymerization reaction was not fully completed under this curing condition. After exposure to 300 °C, distinct dissolution features appeared on the surfaces of the fly ash particles. Partially reacted structures formed, suggesting improved contact between the binder phase and the particles. In specimens exposed to 600 °C, the N–A–S–H gel phase became more dominant; however, microcracks and an irregular porous structure were also evident. After exposure to 900 °C, the appearance of a pronounced interfacial transition zone (ITZ) around the aggregate particles and the interfacial discontinuities between the binder phase and the aggregates implied that, despite matrix reorganization at high temperature, complete structural integrity could not be achieved.

For the H3-0.75 specimen, the fibers were well embedded in the matrix after curing at 90 °C for 48 h, forming a heterogeneous microstructure with fiber-oriented features. Following exposure to 300 °C, microcracks developed around the fibers; however, the fibers clearly bridged these cracks and limited their propagation. At 600 °C, partial degradation of the fibers resulted in more pronounced porous regions surrounding the fiber traces, although fiber residues were still detectable within the matrix. After exposure to 900 °C, the binder phase exhibited a denser structure with a “condensed gel” character, and partial pore closure led to a more compact microstructure. This behavior can be attributed to matrix sintering and the reorganization of glassy phases at high temperature.

Based on these findings, the SEM observations reveal that applying elevated temperatures after 48 h of curing at 90 °C led to a microstructure evolving through successive stages of dissolution, pore formation, and re-densification. While microcracks and porosity were dominant in the 300–600 °C range, matrix reorganization and the formation of a more compact gel phase were observed at 900 °C. In the hemp fiber-reinforced specimens, the fibers played a significant role in restricting microcrack propagation at intermediate temperatures, and even after fiber degradation, the remaining voids were partially compensated by the reorganization of the gel phase at high temperatures. These microstructural findings are in good agreement with the mechanical performance trends observed in the high-temperature tests and confirm that hemp fibers make a meaningful, albeit indirect, contribution to the high-temperature behavior of lightweight geopolymer mortars.

### 3.9. Energy Dispersive X-Ray Spectroscopy Analysis (EDX)

**Figure 14:**
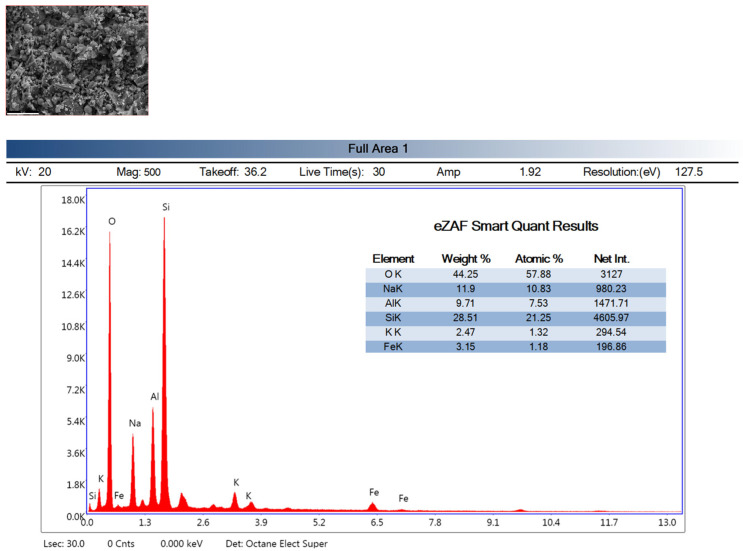
EDX analysis of the reference mixture.

**Figure 15:**
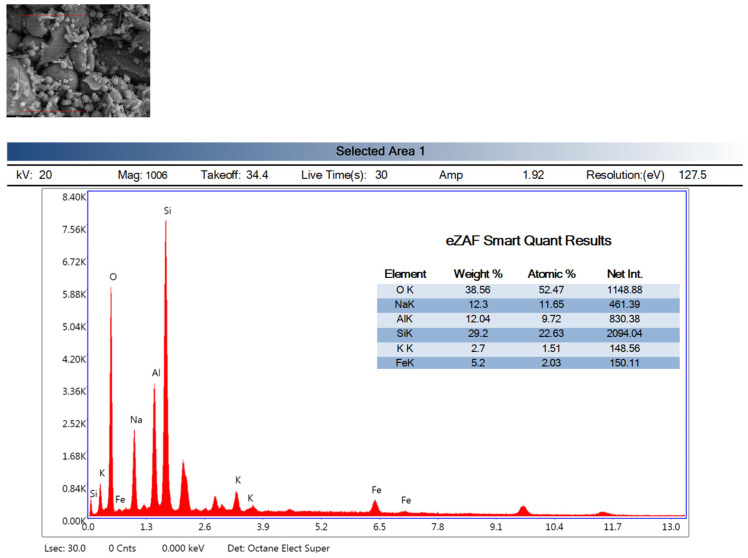
EDX analysis of the H3-0.75.

The EDX analyses of the reference (Ref) and H3-0.75 specimens heat-cured at 90 °C for 48 h heat curing are presented in Figure 14 and Figure 15, respectively. The high contents of O, Si, and Al in the Ref specimen (O: 44.25 wt%, Si: 28.51 wt%, Al: 9.71 wt%) indicate the formation of the typical N-A-S-H gel structure expected in fly ash–based geopolymer systems. The Na content of 11.90 wt% confirms that the alkaline activation process was effectively achieved and that sodium ions were successfully incorporated into the gel network. The relatively lower contents of K (2.47 wt%) and Fe (3.15 wt%) suggest that fly ash-derived secondary phases and mineral impurities remained in limited amounts within the matrix. A very similar chemical distribution was also obtained for the H3-0.75 specimen, with O, Si, Al, and Na contents of 38.56 wt%, 29.20 wt%, 12.04 wt%, and 12.30 wt%, respectively. These values demonstrate that the fundamental elemental composition required for N-A-S-H gel formation in aluminosilicate-based geopolymer systems was preserved in the H3-0.75 mixture. The comparable Si and Al contents between the Ref and H3-0.75 specimens indicate that the gel network exhibits a similar chemical character in both mixtures. Likewise, the close Na contents confirm that the alkaline activation mechanism remained effective in both systems. The presence of K (2.70 wt%) and Fe (5.20 wt%) further indicates that fly ash-derived mineral components continued to be incorporated into the matrix of the H3-0.75 specimen. In summary, the EDX results reveal that the Ref and H3-0.75 specimens exhibit a high degree of similarity in chemical composition and that both systems provide a suitable chemical environment for the formation of N-A-S-H gel.

### 3.10. The Elemental Mapping Results

**Figure 16:**
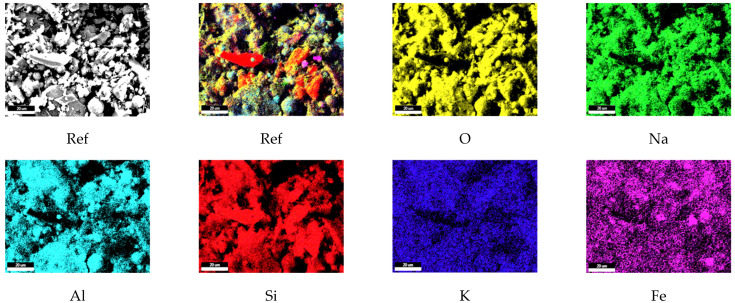
The elemental mapping results of the reference (Scale bar: 20 µm).

**Figure 17:**
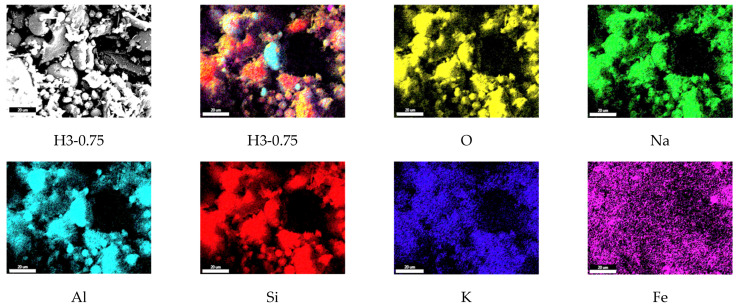
The elemental mapping results of the H3-0.75 (Scale bar: 20 µm).

The elemental mapping results for the reference (Ref) and H3-0.75 specimens cured at 90 °C for 48 h are presented in Figure 16 and Figure 17. In both the Ref and H3-0.75 mixtures, O, Al, and especially Si exhibit a partially homogeneous distribution over vast areas, indicating that the silica-rich environment created by the combined use of fly ash and perlite provides a suitable basis for geopolymerization. The high-intensity, continuous distribution of silicon confirms that the silica-rich nature of the perlite aggregate supports the formation of a three-dimensional polymeric network. The overlapping distributions of Al and Si further demonstrate that the fundamental components of the N-A-S-H gel structure coexist within the matrix.

The Na and K elements display lower but widely distributed intensities, suggesting that the alkaline activators are effectively incorporated into the gel structure and function as charge-balancing ions. In contrast, the Fe element is concentrated in more localized and limited regions, corresponding to fly ash-derived secondary phases and representing filler-type regions rather than the primary binding phase.

### 3.11. X-Ray Diffraction Analysis (XRD)

**Figure 18:**
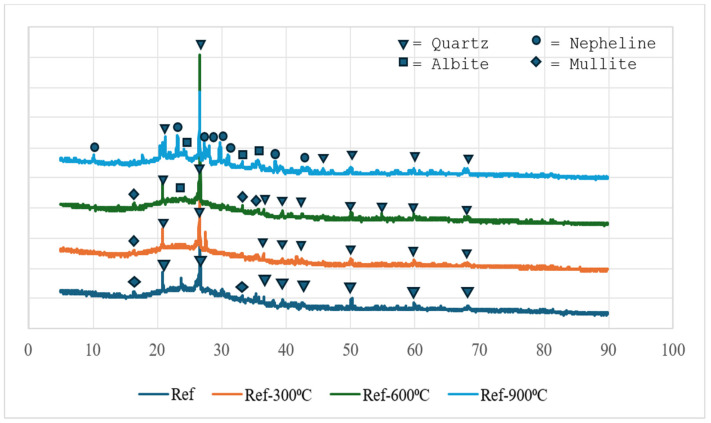
XRD results of reference.

**Figure 19:**
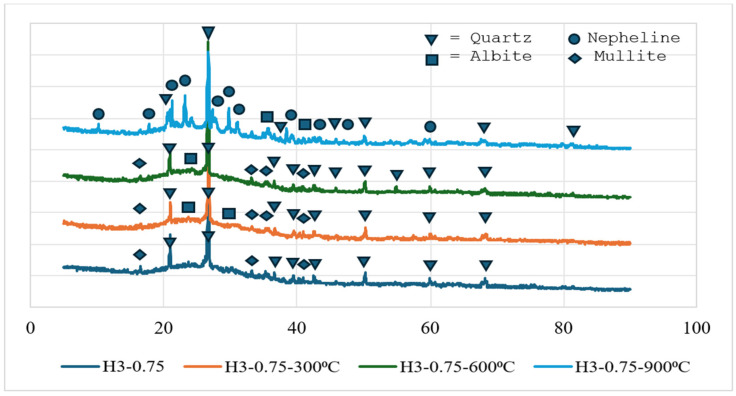
XRD results of H3-0.75.

The XRD analysis results for the reference (Ref) and H3-0.75 samples cured at 90 °C for 48 h are shown in Figure 18 and Figure 19, respectively. Examination of the diffraction patterns reveals that significant phase transformations occur in the geopolymer matrix as temperature increases. At 90 °C and 300 °C, the patterns are mainly characterized by quartz and mullite peaks, indicating that these phases remain as crystalline components originating from the fly ash and perlite aggregates. The appearance of albite peaks above 300 °C suggests that Na-rich aluminosilicate phases become more pronounced at this temperature. At 600 °C, quartz, mullite, and albite phases coexist, while the gradual reduction in the amorphous hump indicates the progressive transformation of the geopolymer gel into crystalline phases. After exposure to 900 °C, nepheline peaks are observed for the first time. This phase represents a thermally stable, high-temperature product formed by the recrystallization of a Na-rich aluminosilicate gel, and, together with mullite and quartz, it points to the evolution of the system toward a more compact, thermally stable microstructure. The increase in compressive and flexural strength observed after 900 °C can be directly attributed to the formation of the nepheline phase, reflecting the rearrangement of the geopolymer gel into a denser, mechanically stronger crystalline framework at elevated temperatures. Overall, the XRD findings demonstrate that the geopolymer system initially consists of amorphous gel accompanied by quartz and mullite residues at lower temperatures, while the progressive formation of albite and ultimately nepheline phases with increasing temperature leads to a microstructure that is more stable and mechanically resistant.

Geopolymer gels, particularly N-A-S-H type binding phases, are known to be predominantly amorphous in nature and therefore cannot be directly identified by sharp diffraction peaks in XRD patterns. In this context, the geopolymerization process is not evaluated solely based on the formation of new crystalline phases, but also through changes observed in the amorphous region of the diffractograms, such as the presence of a broad diffuse band (amorphous hump) and variations in the intensity of precursor-related peaks. The XRD results obtained in this study are consistent with the typical amorphous structural behavior widely reported for fly ash-based geopolymer systems.

### 3.12. Life Cycle Assessment (LCA)

The life cycle assessment (LCA) results for the reference (Ref) and H3-0.75 mixtures, calculated using a functional unit of 1 m^3^/MPa, are presented in Table 6. This functional unit normalizes the environmental impacts with respect to mechanical performance, enabling a performance-based comparison of the mixtures. The results reveal that the H3-0.75 mixture exhibits lower environmental impact values than the reference mortar in all evaluated impact categories, demonstrating a more sustainable environmental performance. The global warming potential (GWP) of H3-0.75 decreased by approximately 21% to 33.0 kg CO_2_-eq, implying that the combined use of hemp fiber and perlite can significantly reduce the carbon footprint of geopolymer mortars.

Similarly, reductions of about 20–22% were observed in abiotic depletion (AD) and fossil fuel depletion (AD-FF) categories, confirming that the H3-0.75 mixture is more efficient in terms of energy and raw material consumption. Decreases ranging from approximately 19% to 24% in human toxicity (HT), freshwater and marine ecotoxicity (FAE and MAE), and terrestrial ecotoxicity (TE) categories further demonstrate the lower potential adverse impacts of this mixture on human health and ecosystems.

Consistent reductions were also recorded in acidification potential (AP), photochemical oxidation (PO), and eutrophication potential (EP) categories in favor of the H3-0.75 mixture. Although the improvement in the EP category was relatively more limited compared to the others, the overall trend clearly confirms a systematic reduction in environmental burden.

Overall, the LCA findings reveal that the H3-0.75 mixture offers not only superior mechanical and high-temperature performance but also a significantly enhanced environmental sustainability profile compared to the reference mixture. These results highlight that the hemp fiber–perlite combination provides a strong environmental advantage in the design of lightweight geopolymer mortars.

**Figure 20:**
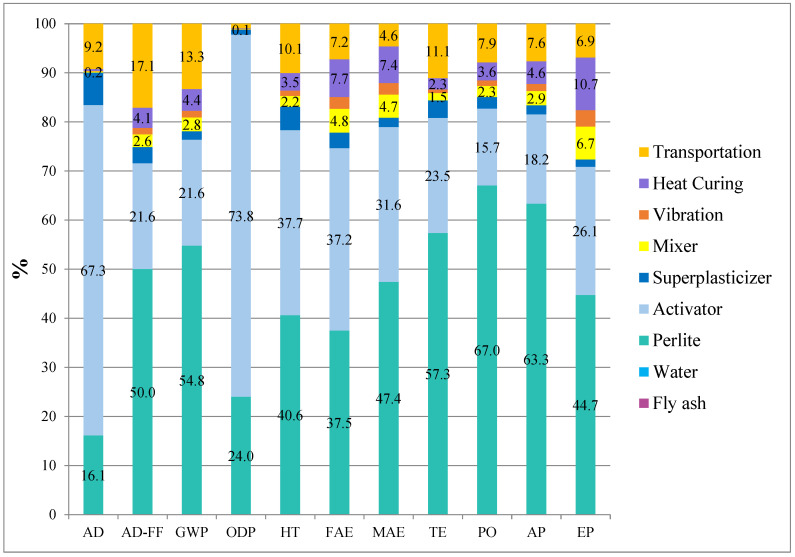
The distribution of environmental impacts of the mortar mixture by sub-components for reference.

**Figure 21:**
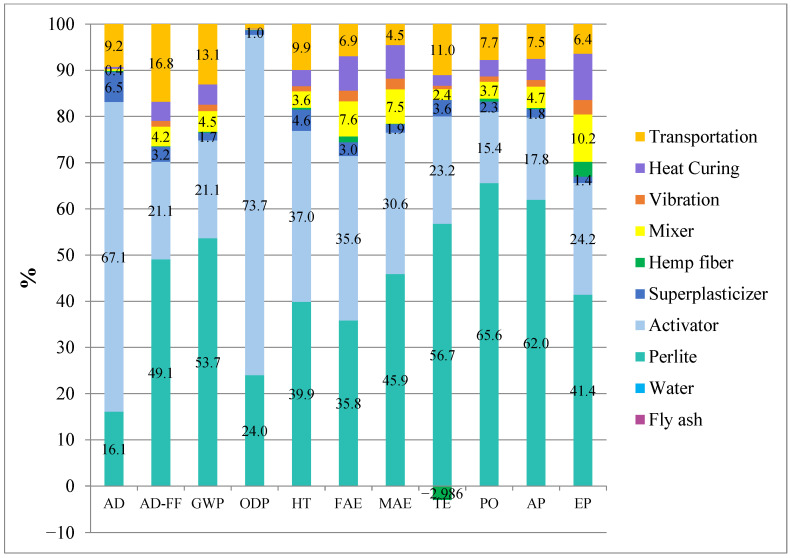
The distribution of environmental impacts of the mortar mixture by sub-components for H3-0.75.

The distribution of environmental impacts for the subcomponents of the reference mortar and the H3-0.75 mixture is shown in Figure 20 and Figure 21. Figure 20 presents the normalized subcomponent contributions of the reference (Ref) mixture obtained from the LCA analysis. The results clearly indicate that the environmental impacts originate predominantly from the activator (NaOH) and perlite production, depending on the impact category. In particular, the activator is the dominant contributor in the AD and ODP categories (67.3 and 73.8, respectively), demonstrating its significant influence on resource depletion and ozone depletion potential. In contrast, perlite contributes the most to the GWP, PO, and AP categories (54.8, 67.0, and 63.3, respectively), highlighting the energy-intensive nature of perlite processing and its associated emissions. Similarly, in the AD-FF category, perlite remains the primary contributor (50.0), showing its substantial impact on fossil fuel depletion. For toxicity-related indicators, both perlite and the activator play comparably significant roles. For example, in the HT category, perlite and the activator contribute 40.6 and 37.7, respectively, while in the FAE category, their contributions are 37.5 and 37.2, respectively. These results confirm that both components substantially influence human and ecological toxicity indicators. Other processes, such as heat curing, transportation, and partially mixer and vibration operations, also provide secondary but non-negligible contributions. Notably, heat curing (10.7) and mixer operations (6.7) have a pronounced effect in the EP category, while transportation shows a considerable impact in the TE category (11.1).

Figure 21 reports the normalized subcomponent contributions of the H3-0.75 mixture derived from the LCA subcomponent analysis. Similarly to the reference system, the results show that environmental burdens are mainly determined by the activator (NaOH) and perlite. At the same time, the relative importance of secondary processes varies across impact categories. The activator remains the dominant hotspot for AD and ODP (67.059 and 73.707, respectively), confirming that alkali activation is the primary driver of resource-depletion- and ozone-depletion-related impacts. In contrast, perlite is the leading contributor to GWP, PO, and AP (53.654, 65.581, and 61.952), indicating that the energy- and processing-related emissions associated with perlite production essentially control climate-change and air-pollution–related categories. Perlite also dominates AD-FF (49.051), highlighting its substantial role in fossil fuel depletion.

For toxicity-related indicators, both perlite and the activator contribute strongly, often at comparable levels (e.g., HT: perlite 39.875 vs. activator 37.010; FAE: perlite 35.831 vs. activator 35.590), suggesting that these two inputs jointly shape the overall toxicity and ecotoxicity profiles of the mixture. Regarding the remaining processes, transportation shows a noticeable contribution, particularly in TE (11.004) and also across several categories (e.g., AD-FF 16.819; GWP 13.065). Moreover, heat curing and process energy-related steps (mixer and vibration) become more pronounced in some categories; notably, EP is primarily influenced by mixer and heat curing (10.228 and 10.004), with additional contribution from vibration (3.143). The contribution of hemp fiber is generally minor across most categories; however, the negative contribution observed in TE (−2.986) suggests a potential credit effect within the inventory/model assumptions (e.g., biogenic carbon uptake or avoided burdens). It should therefore be interpreted cautiously within the boundaries of the applied LCA framework.

Overall, the subcomponent-based LCA analysis indicates that both the reference and H3-0.75 mixtures can achieve significant environmental improvement primarily through optimization of the activator and perlite supply chains. Transportation and curing-related energy consumption also represent secondary but relevant targets for further environmental optimization.

### 3.13. Further Discussions

#### 3.13.1. Mechanical and Microstructural Mechanisms

The observed changes in the physical and mechanical properties of the developed geopolymer mortars can be primarily attributed to microstructural modifications induced by both the expanded perlite aggregate and hemp fiber incorporation. The use of expanded perlite, characterized by its highly porous structure and low density, significantly altered the internal pore network of the composites [33,65]. This resulted in reduced unit weight and modified ultrasonic pulse velocity behavior, which are strongly linked to pore structure continuity and matrix compactness [66]. The incorporation of hemp fibers further influenced the microstructure by introducing additional interfaces within the matrix. At moderate fiber contents, the fibers contributed to crack-bridging effects and improved stress transfer mechanisms, which are reflected in the enhanced flexural and compressive strength values [34,43]. However, the presence of fibers also increased the overall surface area and internal friction, thereby affecting fresh-state flowability. FESEM observations revealed a relatively continuous and compact gel matrix along with partially reacted particles, supporting the interpretation that microstructural densification and matrix continuity play a critical role in governing the macroscopic behavior of the composites [33].

#### 3.13.2. Environmental Assessment

The environmental motivation of the study is supported by the life cycle assessment (LCA) results, which demonstrate that the incorporation of hemp fibers contributes to improved environmental performance relative to the reference mixture. The use of fly ash as the primary binder phase inherently reduces the clinker-related carbon footprint, while the replacement of conventional dense aggregates with expanded perlite further decreases material-related environmental burdens [3,11,16]. Moreover, the bio-based nature of hemp fibers introduces an additional sustainability advantage, particularly in terms of reduced global warming potential. When evaluated from a broader perspective, the developed composites offer a favorable trade-off between mechanical performance, density reduction, and thermal insulation capability. This is found to be parallel in the literature since lower density corresponds to better thermal insulation [67]. This multi-criteria performance profile indicates that such materials may represent viable alternatives to conventional insulation-oriented construction materials. The integration of environmental considerations with engineering performance highlights the relevance of geopolymer-based lightweight composites within sustainable construction frameworks.

#### 3.13.3. Applications, Prospects, and Limitations

Although the developed geopolymer composites do not exhibit strength levels comparable to structural concretes, their reduced unit weight and favorable thermal insulation characteristics suggest promising potential for non-structural applications. In particular, the combination of lightweight expanded perlite aggregate and bio-based hemp fibers enables the production of materials suitable for insulation panels, partition elements, and building envelope components where low density and thermal performance are prioritized over high load-bearing capacity. Excessive fiber length and dosage were observed to limit flowability, indicating that mixture design parameters must be adjusted depending on the target application. Future performance improvements may be achieved through optimizing fiber dispersion, modifying curing regimes, or employing alternative activator compositions. These strategies may enhance matrix homogeneity and interfacial bonding, thereby improving both mechanical and durability-related properties.

## 4. Conclusions

The results of this study confirm that expanded perlite aggregate is highly effective for producing lightweight geopolymer mortars with very low unit weight and favorable thermal insulation characteristics. The physical and mechanical behavior of the mortars was strongly influenced by hemp fiber incorporation, with both fiber length and dosage playing a critical role. Unit weight decreased with increasing heat-curing duration and stabilized after prolonged curing, while ultrasonic pulse velocity measurements indicated that intermediate fiber lengths better preserved microstructural continuity. In terms of mechanical performance, a moderate fiber dosage yielded the most balanced behavior, whereas excessive fiber content led to strength reductions due to fiber agglomeration and increased water demand. Overall, properly proportioned hemp fiber reinforcement enhanced both flexural and compressive responses of the lightweight geopolymer system.

Elevated temperature exposure revealed a temperature-dependent performance evolution typical of geopolymer matrices. Although mechanical strengths decreased at intermediate temperatures, substantial recovery was observed at higher temperatures, which was attributed to matrix sintering and microstructural reorganization. Microstructural investigations supported these findings, showing that fibers contributed to crack-bridging mechanisms at moderate temperatures, while matrix densification and phase transformations governed high-temperature behavior. The formation of thermally stable crystalline phases at elevated temperatures was consistent with the observed strength recovery. From an environmental perspective, life cycle assessment results demonstrated that hemp fiber incorporation improved the sustainability profile of the mortars, while subcomponent analyses identified the activator and perlite production as the primary contributors to environmental impacts. Collectively, these findings indicate that the combined use of hemp fibers and expanded perlite offers a promising pathway for developing lightweight, thermally efficient, and environmentally sustainable geopolymer composites suitable for non-structural applications.

## Figures and Tables

**Table 1:** Chemical Composition of Fly Ash.

Oxides	SiO_2_	Al_2_O_3_	Fe_2_O_3_	K_2_O	Na_2_O	MgO	CaO	SO_3_	Cl	Free CaO
Value (%)	51.7	26.1	6.9	4.55	0.64	1.69	2.75	0.33	0.0043	0.02

**Table 2:** Chemical composition of alkali activator.

Material	NaOH (%)	Na_2_CO_3_ (%)	Cl (%)	Fe(%)	Al (%)	SO_4_ (%)
NaOH	99.09	0.89	0.008	0.0001	0.001	0.008

**Table 3:** Mix proportions.

Mix Code	Na^+^ Ratio (%)	Fly Ash (g)	Perlite (g)	Water (g)	Hemp Fiber (%)	SP (g)
Reference	10	450	220	105	0	6
H1-0.50	10	450	220	115	0.50	6
H1-0.75	10	450	220	125	0.75	6
H1-1.00	10	450	220	135	1.00	6
H2-0.50	10	450	220	115	0.50	6
H2-0.75	10	450	220	125	0.75	6
H2-1.00	10	450	220	135	1.00	6
H3-0.50	10	450	220	115	0.50	6
H3-0.75	10	450	220	125	0.75	6
H3-1.00	10	450	220	135	1.00	6

**Table 4:** Weight loss after the elevated temperature test.

Mix Code	Weight Loss After 300 °C (%)	Weight Loss After 600 °C (%)	Weight Loss After 900 °C (%)
Ref	4.88	7.01	8.18
H1-0.75	4.24	7.11	7.36
H2-0.75	4.54	7.76	8.25
H3-0.75	4.71	7.93	8.75

**Table 5:** UPV values after the elevated temperature test.

Mix Code	UPV Before Test (km/s)	UPV After 300 °C (km/s)	UPV After 600 °C (km/s)	UPV After 900 °C (km/s)
Ref	2.15	1.7	1.53	3.62
H1-0.75	2.19	1.74	1.55	3.67
H2-0.75	2.15	1.7	1.52	3.68
H3-0.75	2.32	1.81	1.59	3.62

**Table 6:** LCA results of specimens.

Category	Unit	Ref	H3-0.75
AD	kg Sb eq	1.94 × 10^−4^	1.51 × 10^−4^
AD-FF	MJ	4.54 × 10^2^	3.60 × 10^2^
GWP	kg CO_2_ eq	4.16 × 10^1^	3.30 × 10^1^
ODP	kg CFC-11 eq	7.57 × 10^−6^	5.88 × 10^−6^
HT	kg 1,4-DB eq	2.68 × 10^1^	2.11 × 10^1^
FAE	kg 1,4-DB eq	1.61 × 10^1^	1.30 × 10^1^
MAE	kg 1,4-DB eq	5.01 × 10^4^	4.02 × 10^4^
TE	kg 1,4-DB eq	1.74 × 10^−1^	1.33 × 10^−1^
PO	kg C_2_H_4_ eq	1.12 × 10^−2^	8.92 × 10^−3^
AP	kg SO_2_ eq	2.39 × 10^−1^	1.91 × 10^−1^
EP	kg PO_4_ eq	6.70 × 10^−2^	5.62 × 10^−2^

## Data Availability

The data are available from the corresponding author upon reasonable request, due to institutional restrictions.
